# Polymorphisms in *TS*, *MTHFR* and *ERCC1* genes as predictive markers in first-line platinum and pemetrexed therapy in NSCLC patients

**DOI:** 10.1007/s00432-014-1756-6

**Published:** 2014-07-16

**Authors:** Paweł Krawczyk, Tomasz Kucharczyk, Dariusz M. Kowalski, Tomasz Powrózek, Rodryg Ramlau, Ewa Kalinka-Warzocha, Kinga Winiarczyk, Magdalena Knetki-Wróblewska, Kamila Wojas-Krawczyk, Katarzyna Kałakucka, Wojciech Dyszkiewicz, Maciej Krzakowski, Janusz Milanowski

**Affiliations:** 1 Department of Pneumonology, Oncology and Allergology, Medical University of Lublin, Jaczewskiego 8, 20-954 Lublin, Poland; 2 Postgraduate School of Molecular Medicine, Warsaw Medical University, Warsaw, Poland; 3 Department of Lung and Chest Cancer, Oncology Centre - Institute M. Sklodowska-Curie, Warsaw, Poland; 4 Wielkopolska Center of Pulmonology and Thoracosurgery of Eugenia and Janusz Zeyland, Poznan, Poland; 5 Department of Thoracic Surgery, Poznan University of Medical Sciences, Poznan, Poland; 6 Regional Centre of Oncology, Lodz, Poland; 7 Institute of Agricultural Medicine of Lublin, Lublin, Poland

**Keywords:** Non-small cell lung cancer, Pemetrexed, Platinum compounds, Gene polymorphisms

## Abstract

**Purpose:**

We presented retrospective analysis of up to five polymorphisms in *TS*, *MTHFR* and *ERCC1* genes as molecular predictive markers for homogeneous Caucasian, non-squamous NSCLC patients treated with pemetrexed and platinum front-line chemotherapy.

**Methods:**

The following polymorphisms in DNA isolated from 115 patients were analyzed: various number of 28-bp tandem repeats in 5′-UTR region of *TS* gene, single nucleotide polymorphism (SNP) within the second tandem repeat of *TS* gene (G>C); 6-bp deletion in 3′-UTR region of the *TS* (1494del6); 677C>T SNP in *MTHFR*; 19007C>T SNP in *ERCC1*. Molecular examinations’ results were correlated with disease control rate, progression-free survival (PFS) and overall survival.

**Results:**

Polymorphic tandem repeat sequence (2R, 3R) in the enhancer region of *TS* gene and G>C SNP within the second repeat of 3R allele seem to be important for the effectiveness of platinum and pemetrexed in first-line chemotherapy. The insignificant shortening of PFS in 3R/3R homozygotes as compared to 2R/2R and 2R/3R genotypes were observed, while it was significantly shorter in patients carrying synchronous 3R allele and G nucleotide. The combined analysis of *TS* VNTR and *MTHFR* 677C>T SNP revealed shortening of PFS in synchronous carriers of 3R allele in *TS* and two C alleles in *MTHFR*. The strongest factors increased the risk of progression were poor PS, weight loss, anemia and synchronous presence of 3R allele and G nucleotide in the second repeat of 3R allele in *TS*. Moreover, lack of application of second-line chemotherapy, weight loss and poor performance status and above-mentioned genotype of *TS* gene increased risk of early mortality.

**Conclusion:**

The examined polymorphisms should be accounted as molecular predictor factors for pemetrexed- and platinum-based front-line chemotherapy in non-squamous NSCLC patients.

## Introduction

Lung cancer is the main cause of death among all cancer patients in the world. Over 85 % of such cases are represented by non-small cell lung cancer (NSCLC) and only 15 % of those cases have a chance of surgical resection (Dela Cruz et al. 2011). This is why systemic chemotherapy and radiotherapy are the main treatment options in NSCLC.

Standard first-line chemotherapy consisting of platinum compounds and drugs like gemcitabine or vinorelbine, used in NSCLC therapy, has proven to be effective, but at a cost of sometimes serious side effects (Sculier and Moro-Sibilot 2009). Therefore, new 3rd generation drugs (pemetrexed) with lower toxicity profiles are being developed and used in combination with platinum compounds in first-line treatment of non-squamous NSCLC patients (Scagliotti et al. 2008).

Platinum compounds are the alkylating agents, which cross-link DNA in several different ways interfering with transcription and cell division. The damaged DNA elicits DNA repair mechanisms or activates apoptosis when repair proves impossible. Excision repair cross-complementing group 1 (ERCC1) is an endonuclease, which is involved in DNA repair in nucleotide excision repair mechanism (NER). High expression of ERCC1 and other NER enzymes is associated with a high yield of DNA repair. Increased DNA repair is associated with longer survival of surgically treated patients, but is negative predictive factor for chemotherapy using platinum compounds (Zheng et al. 2007; Gazdar 2007).

Pemetrexed is a novel cytostatic drug, which has shown efficacy in first and second line of treatment, as well as in maintenance therapy, of non-squamous NSCLC and malignant pleural mesothelioma (Scagliotti et al. 2008, 2009; Al-Saleh et al. 2012). Pemetrexed is a multitarget antifolate agent that inhibits enzymes involved in pyrimidine and purine synthesis. Its main target is thymidylate synthase (TS). Inhibition of TS results in decreased amount of thymidyne, which is necessary for DNA repair and synthesis. Pemetrexed also inhibits two other enzymes involved in purine/pyrimidine synthesis pathway: dihydrofolate reductase (DHFR) and glycinamide ribonucleotide formyltransferase (GARFT). It is also known that the activity of those target enzymes alters the effectiveness of pemetrexed. High expression of TS, DHFR and GARFT in squamous cell lung cancer and some of lung adenocarcinoma was proven to reduce chemosensitivity of cancer cells to pemetrexed, and pemetrexed was inefficient in patients with this histologic subtype of NSCLC (Scagliotti et al. 2009; Chattopadhyay et al. 2007; Hanauske et al. 2007; Wang et al. 2014; Ceppi et al. 2006).

All three enzymes are folate dependent, and hence, the activity of pemetrexed also depends on cell folate level. It has been shown that increased levels of extracellular folates decreased pemetrexed activity in human lung and colon cancer cell lines (Chattopadhyay et al. 2007). Enzyme that is indirectly involved in the proper functioning of purine and pyrimidine synthesis pathway is 5,10-methylenetetrahydrofolate reductase (MTHFR). It catalyzes an irreversible conversion of 5,10-methylenetetrahydrofolate (5,10-methyleneTHF) to methyltetrahydrofolate (5-methylTHF). Elevated levels of 5,10-methyleneTHF causes higher activity of TS and lower effectiveness of antifolate agents in cancers patients (Tiseo et al. 2012).

Thymidylate synthase mRNA level is regulated by three different polymorphisms: various number of 28-base-pair (bp) tandem repeats (VNTR) in 5′-UTR enhancer region of *TS* gene (*TSER*) (Mandola et al. 2003); a single nucleotide polymorphism (SNP) G>C in the second repeat of 28-bp repeats; and a 6-bp deletion on the 3′ end of the *TS* gene (1494del6) (Kawakami and Watanabe 2003; Uchida et al. 2004; Mandola et al. 2004; Stoehlmacher et al. 2009). VNTR and 1494del6 polymorphisms of *TS* gene are known to affect the effectiveness of 5-fluorouracil treatment of metastatic colorectal cancer and breast cancer patients (Dotor et al. 2006; Kumar et al. 2010). A polymorphism 677C>T in *MTHFR* gene was found to decrease the gene expression and hence to cause elevated levels of 5,10-methyleneTHF (Sohn et al. 2013). Several authors demonstrated that 19007C>T (Asn118Asn) polymorphism of *ERCC1* gene is involved in ability to DNA repair. However, correlation between genotypes of *ERCC1* gene and the expression of ERCC1 protein in cancer cells is still controversial (Mlak et al. 2013).

In our retrospective, non-randomized, multicenter study, we tried to assess the usefulness of *TS*, *MTHFR* and *ERCC1* genes polymorphisms detected in venous blood cells as molecular predictive markers for first-line pemetrexed and platinum therapy in NSCLC patients.

## Materials and methods

The studied group consisted of 115 non-squamous NSCLC patients treated with pemetrexed in combination with platinum compounds (cisplatin in 108 patients, carboplatin in 7 patients). All patients have been verified pathomorphologically and staged by computed tomography. Medical history data of each patient were collected. Patients’ performance status was assessed according ECOG-WHO score. Patients were qualified for the therapy between 2008 and 2012 year. About 500 mg/m^2^ of pemetrexed was administered as an intravenous infusion on day 1 of each 21 day cycle. Cisplatin 75 mg/m^2^ infused over 2 h beginning approximately 30 min after the end of the pemetrexed administration. Carboplatin was dosed according to New Guidelines for Carboplatin Dosing. To reduce toxicity, patients were pretreated with folic acid and B12 vitamin. Clinical characteristic of patients is presented in Table 1.

**Table 1:** Characteristic of studied group

Factor	Characteristics
Whole group (*n*)	115
Gender	
Male (*n*, %)	59 (51.3)
Female (*n*, %)	56 (48.7)
Age in years (median ± SD)	61 ± 8.5
≤61 years (*n*, %)	50 (43.5)
>61 years (*n*, %)	65 (56.5)
Performance status (PS)	
PS = 0 (*n*, %)	38 (33)
PS = 1 (*n*, %)	77 (67)
Weight loss	
<5 % (*n*, %)	61 (53)
≥5 % (*n*, %)	54 (47)
Anemia	
Yes (*n*, %)	42 (36.5)
No (*n*, %)	73 (63.5)
Smoking status	
Smokers (*n*, %)	72 (62.6)
Non-smokers (*n*, %)	21 (18.3)
Data not available (*n*, %)	22 (19.1)
Disease stage	
Inoperable (II–IIIB) (*n*, %)	29 (25.2)
Advanced (IV) (*n*, %)	86 (74.8)
Pathomorphological diagnosis	
Adenocarcinoma (*n*, %)	106 (92.2)
Large cell carcinoma (*n*, %)	8 (7)
Adenosquamous cell carcinoma (*n*, %)	1 (0.8)
Prior surgical treatment	
Yes (*n*, %)	26 (22.6)
No (*n*, %)	89 (77.4)
Radiotherapy	
Yes (*n*, %)	22 (19.1)
No (*n*, %)	93 (80.9)
No. of first-line chemotherapy cycles (median, range)	4, 1–9
First-line chemotherapy	
Cisplatin + pemetrexed (*n*, %)	108 (93.9)
Carboplatin + pemetrexed (*n*, %)	7 (6.1)
Second-line chemotherapy	
Docetaxel (*n*, %)	10 (8.7)
Paclitaxel (*n*, %)	8 (7)
Erlotinib (*n*, %)	4 (3.5)
Crizotinib (*n*, %)	2 (1.7)
Belagenpumatucel-L (*n*, %)	2 (1.7)
Vinorelbine (*n*, %)	5 (4.3)
Carboplatin + vinorelbine (*n*, %)	1 (0.9)
Gemcitabine (*n*, %)	4 (3.5)
Cisplatin + gemcitabine (*n*, %)	2 (1.7)
Pemetrexed (*n*, %)	3 (2.6)
None (*n*, %)	74 (64.3)
Third-line therapy	
Docetaxel (*n*)	1
Gemcitabine (*n*)	1
Eribulin (*n*)	1
Erlotinib (*n*)	1

Response to chemotherapy has been described according to RECIST criteria. During observation period (2008–February 2014), progression was observed in 85 patients and 57 patients died (complete observations).

Blood samples of examined patients have been collected in four oncology centers in Poland. All molecular examinations have been performed in one clinical laboratory in Pneumonology, Oncology and Allergology Department in Lublin. Five milliliters of blood samples have been collected in EDTA-covered tubes. DNA was extracted from peripheral blood cells using QIAamp DNA Mini Kit (Qiagen, Germany). This study has been approved by the Ethics Committee of Medical University in Lublin (No. KE-0254/219/2010).

### *TS* and *MTHFR* genotyping

*TS* gene VNTR polymorphism was analyzed by polymerase chain reaction (PCR) using primers previously described by Iacopetta et al. (2001) with further modification. The 6-bp deletion on 3′ end of the *TS* gene and the SNP 677C>T in *MTHFR* gene were analyzed with PCR amplification followed by restriction fragment length polymorphism analysis (RFLP). Primers for 1494del6 were previously described by Dotor et al. (2006), whereas *MTHFR* primers were described by Frosst et al. (1995). Restriction enzymes used in RFLP reaction were Fermentas FastDigest *DraI* (Thermo Scientific, USA) for *TS* 1494del6 polymorphism and Fermentas FastDigest *HinfI* (Thermo Scientific, USA) for *MTHFR* 677C>T SNP.

The polymorphism of *TS* (*TSER*) VNTR differs according to the number of 28-bp repeats and in Caucasian population is most often represented by three genotypes: two repeats/two repeats (2R/2R), three repeats/three repeats (3R/3R) and heterozygous of 2R/3R.

All PCR reaction products were later visualized on 2 % agarose gel with ethidium bromide.

Additionally, Sanger sequencing was used for the validation of the presence of different polymorphic forms of examined genes.

### *ERCC1* genotyping

*ERCC1* SNP (19007C>T) was detected using High Resolution Melt (HRM) method. Amplification of examined region and HRM procedure were performed in 48-well plates using the Eco Real-Time PCR device (Illumina). PCR cycling and HRM conditions were performed according to Precision™ HRM protocol. Before PCR reaction, all examined samples were diluted to the same concentration of DNA (20 ng/µl) for similar amplification of investigated gene region and improved quality of HRM. Additionally, DNA samples with all known allelic variants of 19000C>T were enrolled as HRM controls. EcoStudy Software (Illumina) was used for melt curves analysis. Different genotypes of *ERCC1* were distinguished according to normalization data derived from the raw data plots and difference graph derived from the normalization data. HRM results of ERCC1 SNP analysis was validated by SNaPshot technique.

### Statistical analysis

Chi square test was used to compare the quantity of patients with different response to treatment depending on the prevalence of clinical and genetic factors. To compare the probability of progression-free survival and overall survival between the groups with different clinical and molecular factors, the Kaplan–Meier method was used. Cox regression model with step-by-step selection was used to determine the influence of these factors on progression-free survival (PFS) and overall survival (OS) of patients treated with platinum and pemetrexed therapy.

## Results

The enhancer region of *TS* gene (*TSER*) contains a polymorphic tandem repeat sequence (in our study only 2 or 3 repeats, 2R or 3R). 22.6 % of non-squamous NSCLC patients carried 2R/2R genotype, while 58.3 % of patients were heterozygotes 2R/3R, and 19.1 % of patients were homozygotes 3R/3R. Guanine nucleotide within the second repeat of the 3R alleles was found in 34.8 % of examined patients (G>C polymorphism). Deletion of 6 bp in 1494 position in both alleles of *TS* gene was described in 7 % of patients, but this deletion in only one allele of *TS* gene—in 37.4 % of patients. Rarer T allele in 677C>T *MTHFR* gene polymorphism was observed in 53.9 % of patients. In examined group, there were 15.6 % of C/C genotype carriers, 46.1 % of C/T genotype and 38.3 % of TT genotype of *ERCC1* gene 19007C>T polymorphism.

The partial remission to first-line chemotherapy with platinum compounds and pemetrexed was observed in 29 patients with non-squamous NSCLC (25.2 %). Stable disease was noted in 66 of examined patients (57.4 %) and disease progression in 20 patients (17.4 %). Disease control rate was noted in 82.6 % of patients. For whole group of patients, the median time of progression-free survival was 7 months, while the median time of overall survival was 14 months.

Some common clinical factors strongly affected the course of non-squamous NSCLC in patients treated with platinum compounds and pemetrexed in first-line therapy. Patients in poor performance status had significantly higher incidence of early progression during first 2 month of treatment than patients with good PS. PFS was significantly shorter in patients with anemia, body mass loss and in poor performance status as compared to patients without these factors. Regarding the overall survival of patients treated with platinum and pemetrexed, poor performance status, weight loss, anemia and lack of second-line treatment had a significant negative prognostic value. Stage of disease, gender and age had no impact on prognosis in our patients (Table 2).

**Table 2:** Correlation between clinical factors and risk of early progression, duration of PFS and OS in non-squamous NSCLC patients treated with first-line chemotherapy with platinum compounds and pemetrexed

Factor	No.	PD *n* (%)	SD or PR *n* (%)	*p* *χ* ^2^	Median PFS (months)	*p* *χ* ^2^	HR [95 % CI]	Median OS (months)	*p* *χ* ^2^	HR [95 % CI]
Whole group	115	20 (17.4)	95 (82.6)	–	7	–	–	14	–	–
Age (years)										
≤61 years	65	13 (20)	52 (80)	0.553 0.352	6	0.506 0.442	1.148 [0.7497–1.7574]	14	0.718 0.130	1.103 [0.4842–1.3937]
>61 years	50	7 (14)	43 (86)		7.5			13		
Gender										
Male	59	13 (22)	46 (78)	0.270 1.215	6.5	0.394 0.728	0.838 [0.5476–1.2817]	14	0.461 0.543	0.822 [0.4842–1.3937]
Female	56	7 (12.5)	49 (87.5)		7.5			13		
Smoking status										
Smoker	72	14 (19.4)	58 (80.6)	0.552 0.353	7.5	0.246 1.346	0.726 [0.391–1.3477]	15	0.812 0.057	0.922 [0.4602–1.8460]
Non-smoker	21	6 (28.6)	15 (71.4)		6			12		
Performance status										
PS = 0	38	2 (5.3)	36 (94.7)	0.032 4.618	9	0.000 14.83	2.221 [1.4510–3.3988]	25	0.000 12.21	2.576 [1.5180–4.3708]
PS = 1	77	18 (23.4)	59 (76.6)		5			11		
Weight loss										
>5 %	54	13 (24.1)	41 (75.9)	0.125 2.348	6	0.004 8.432	1.795 [1.1464–2.8098]	11	0.000 12.55	2.416 [1.3783–4.2350]
≤5 %	61	7 (11.5)	54 (88.5)		8			25		
Anemia										
Yes	42	11 (26.2)	31 (73.8)	0.103 2.666	5	0.002 9.594	1.898 [1.1622–3.1003]	9	0.004 8.391	2.123 [1.1685–3.8584]
No	73	9 (12.3)	64 (87.7)		8			17.5		
Disease stage										
Inoperable	29	6 (20.7)	23 (79.3)	0.796 0.067	6	0.921 0.01	0.976 [0.5946–1.6032]	11.5	0.183 1.771	0.679 [0.3581–1.2883]
Advanced (IV)	86	14 (16.3)	72 (83.7)		7			17.5		
Pathomorphological diagnosis										
Adenocarcinoma	107	18	89	0.916 0.011	7	0.093 2.815	1.800 [0.7064–4.5907]	13.5	0.923 0.010	1.045 [0.4105–2.6621]
Large cell carcinoma	8	2	6		5			12		
Prior surgical treatment										
Yes	26	3 (11.5)	23 (88.5)	0.548 0.361	7	0.189 1.727	0.709 [0.4327–1.1604]	28.5	0.329 0.953	0.705 [0.3714–1.3380]
No	89	17 (19.1)	72 (80.9)		7			13		
Radiotherapy										
Yes	22	3 (13.6)	19 (86.4)	0.838 0.042	7	0.195 1.679	0.715 [0.4362–1.1727]	13	0.481 0.496	0.785 [0.415–1.4848]
No	93	17 (18.3)	76 (81.7)		7			14		
Second-line chemotherapy										
Yes	41							21	0.003 8.842	0.447 [0.2633–0.7579]
No	74							12		

**Fig. 1:**
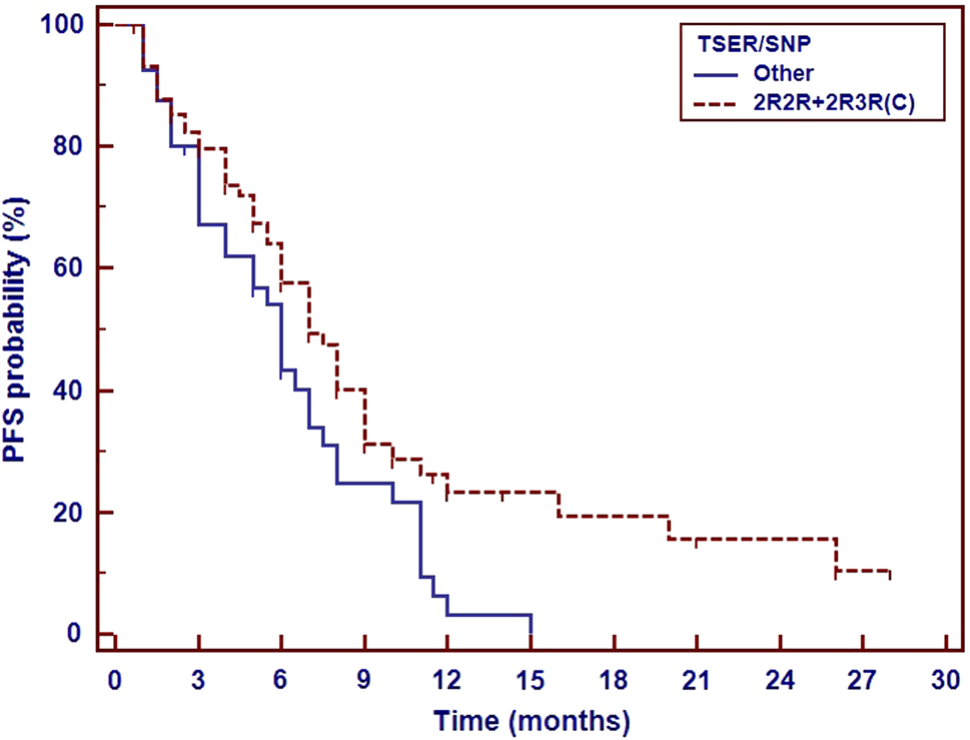
Impact of *TS* VNTR polymorphism and a single-nucleotide polymorphism (G>C) within the second repeat of the 3R allele on the progression-free survival in non-squamous NSCLC patients treated with platinum and pemetrexed first-line therapy

Several polymorphic variants of *TS* and *MTHFR* genes were important as a predictor of platinum compounds and pemetrexed therapy effectiveness in non-squamous NSCLC patients. Polymorphic tandem repeat sequence (2R, 3R) in the enhancer region of *TS* gene (*TSER*) and a single nucleotide polymorphism (G>C) within the second repeat of the 3R allele seem to be important for the effectiveness of this first-line chemotherapy. We observed insignificant shortening of PFS in 3R/3R homozygotes as compared to 2R/2R homozygotes and 2R/3R heterozygotes. PFS was significantly shorter (6 months) in patients carrying synchronous 3R allele and G nucleotide in the second repeat of 3R allele than in carriers of other genotypes of *TS* gene (Fig. 1).

**Fig. 2:**
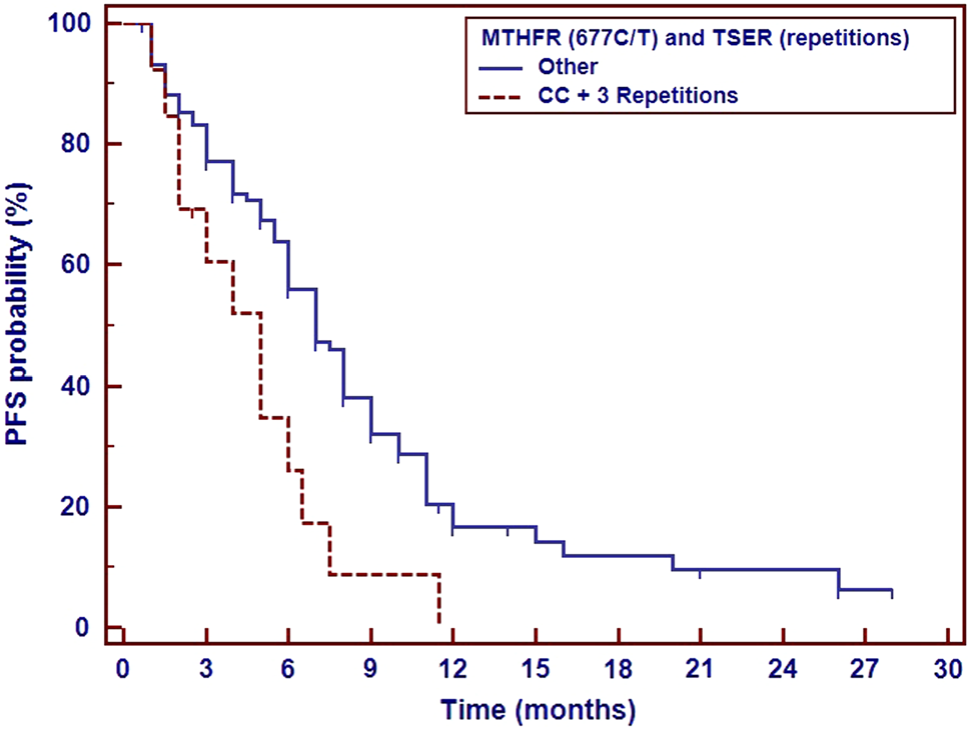
Impact of *TS* VNTR polymorphism and *MTHFR* gene 677C>T polymorphism on the progression-free survival in non-squamous NSCLC patients treated with platinum and pemetrexed first-line therapy

Early disease progression was observed significantly more frequent in patients with T/T genotype than in patients with C/C or C/T genotypes of *MTHFR* 677C>T polymorphism. PFS and OS were not affected by *MTHFR* gene polymorphism. However, combined analysis of *TS* VNTR polymorphism and *MTHFR* 677C>T SNP revealed shortening of PSF (5 months) in synchronous carriers of 3R allele in *TS* gene and two C alleles in *MTHFR* gene (Table 3, Fig. 2).

**Table 3:** *TS*, *MTHFR* and *ERCC1* genes polymorphisms and their impact on the risk of early progression and duration of PFS and OS in non-squamous NSCLC patients treated with first-line chemotherapy with platinum compounds and pemetrexed

Factor	No.	PD *n* (%)	SD or PR *n* (%)	*p* *χ* ^2^	Median PFS (months)	*p* *χ* ^2^	HR [95 % CI]	Median OS (months)	*p* *χ* ^2^	HR [95 % CI]
VNTR of *TS* gene (*TSER*)										
2R/2R	26	2 (7.7)	24 (92.3)	0.476 1.483	7	0.063 5.536	–	11	0.795 0.460	–
2R/3R	67	14 (20.9)	53 (79.1)		7			15		
3R/3R	22	4 (18.2)	18 (81.8)		5.5			13		
VNTR/SNP of *TS* gene (*TSER*)										
2R/2R	26	2 (7.7)	24 (92.3)	0.524 3.207	7	0.019 11.811	–	11	0.505 3.325	–
2R/3R(G)	18	3 (16.7)	15 (83.3)		7			13		
2R/3R(C)	49	11 (22.4)	38 (77.6)		7.5			17.5		
3R(G)/3R(C)	8	3 (37.5)	5 (62.5)		3.5			8		
3R(G)/3R(G)	14	1 (7.1)	13 (92.9)		6			23		
2R/2R + 2R/3R(C)	75	13 (17.3)	62 (82.7)	0.813 0.056	7	0.020 5.445	0.619 [0.3916–0.9798]	14	0.889 0.020	0.963 [0.5638–1.6457]
2R/3R(G) + 3R(C)/3R(G) + 3R(G)/3R(G)	40	7 (17.5)	33 (82.5)		6			13		
1494del6 of *TS* gene										
−/− 6 bp	8	0 (0)	8 (100)	0.131 4.062	9	0.761 0.546	–	–	0.186 3.363	–
+/− 6 bp	43	12 (27.9)	31 (72.1)		6.5			13		
+/+ 6 bp	64	8 (12.5)	56 (87.5)		7			17.5		
+6/+ 6 bp	64	8 (12.5)	56 (87.5)	0.193 1.697	7	0.945 0.005	0.986 [0.6417–1.5139]	12	0.684 0.166	0.899 [0.5322–1.5189]
−6/− 6 bp and +6/− 6 bp	51	12 (23.5)	39 (76.5)		7			14		
677C>T SNP of *MTHFR* gene										
CC	53	9 (17)	44 (83)	0.000 35.07	6	0.840 0.349	–	25	0.227 2.969	–
CT	49	1 (2)	48 (98)		7.5			13		
TT	13	10 (76.9)	3 (23.1)		7			12		
CC	53	9 (17)	44 (83)	0.890 0.019	6	0.573 0.319	1.124 [0.7334–1.7229]	25	0.132 2.265	0.673 [0.3960–1.1428]
CT + TT	62	11 (17.7)	51 (82.3)		7.5			13		
TT	13	10 (76.9)	3 (23.1)	0.000 31.64	7	0.732 0.117	0.885 [0.4426–1.7713]	12	0.745 0.106	0.848 [0.3281–2.1904]
CT + CC	102	10 (9.8)	92 (90.2)		7			14		
677C>T SNP of *MTHFR* gene and VNTR of *TS* gene										
CC + 3R	13	3 (23.1)	10 (76.9)	0.852 0.035	5	0.003 8.783	2.909 [0.8354–10.1301]	13	0.835 0.044	1.093 [0.4549–2.6250]
Other	102	17 (16.7)	85 (82.3)		7			13.5		
19007C>T SNP of *ERCC1* gene										
CC	18	4 (22.2)	14 (77.8)	0.758 0.554	6	0.214 3.083	–	17.5	0.386 1.910	–
CT	53	7 (13.2)	46 (86.8)		8			13		
TT	44	9 (20.5)	35 (79.5)		6			13.5		
CC	18	4 (22.2)	14 (77.8)	0.802 0.063	6	0.136 2.195	0.677 [0.3641–1.2599]	17.5	0.173 1.858	0.658 [0.3259–1.3299]
CT + TT	97	16 (16.5)	81 (83.5)		7			13		
TT	44	9 (20.5)	35 (79.5)	0.668 0.184	6	0.667 0.188	0.913 [0.5887–1.4147]	13.5	0.487 0.483	1.209 [0.7110–2.0563]
CT + CC	71	11 (15.5)	60 (84.5)		7			13		
19007C>T SNP of *ERCC1* gene and 677C>T SNP of *MTHFR* gene										
CC of both genes	8	3 (37.5)	5 (62.5)	0.284 1.149	7	0.331 0.946	1.438 [0.5824–3.5528]	17.5	0.567 0.327	1.273 [0.5007–3.2344]
CT or TT of both genes	107	17 (15.9)	90 (84.1)		7			13		
TT of both genes	6	0 (0)	6 (100)	0.548 0.362	7	0.928 0.001	0.957 [0.3576–2.5592]	13.5	0.802 0.063	0.838 [0.2283–3.0753]
CT or CC of both genes	109	20 (18.3)	89 (81.7)		7			13

Multivariate Cox logistic regression confirmed that the strongest factors that increased the risk of progression in non-squamous NSCLC patients treated with platinum and pemetrexed first-line therapy were poor performance status, weight loss, anemia and synchronous presence of 3R allele and G nucleotide in the second repeat of 3R allele in *TS* gene. Moreover, clinical prognostic factors: lack of application of second-line chemotherapy, weight loss and poor performance status as well as the above-mentioned genotype of *TS* gene increased risk of early mortality (Table 4).

**Table 4:** The multivariate Cox logistic regression of factors affecting the progression-free survival and overall survival of non-squamous NSCLC patients treated with platinum compounds and pemetrexed in first-line chemotherapy

Factor	*β*	*p* value	Hazard ratio [95 % CI]
Progression-free survival			
Poor performance status	0.8504	0.0008	2.3405 [1.4249–3.8444]
Lack of radiotherapy	0.6185	0.0361	1.8561 [1.0439–3.3004]
2R/3R(G) or 3R(C)/3R(G) or 3R(G)/3R(G) of *TS* genotype	0.5043	0.0249	1.6558 [1.0681–2.5668]
Anemia	0.4731	0.046	1.6049 [1.0108–2.5482]
Overall model fit: *p* < 0.0001; *χ* ^2^ = 26,632			
Overall survival			
Lack of second-line treatment	1.1864	0.0008	3.2752 [1.6421–6.5323]
2R/3R(G) or 3R(C)/3R(G) or 3R(G)/3R(G) of *TS* genotype	0.7273	0.022	2.0696 [1.1141–3.8445]
Weight loss <5 %	0.7257	0.0153	2.0662 [1.1525–3.7042]
Poor performance status	0.6534	0.0468	1.9221 [1.0126–3.6486]
Overall model fit: *p* < 0.0001; *χ* ^*2*^ = 29,974			

## Discussion

Pemetrexed with combination of platinum compounds was registered to front-line treatment of non-squamous NSCLC patients based on the large phase III clinical trial (Scagliotti et al. 2008), where patients randomly received either cisplatin plus gemcitabine (*n* = 862) or cisplatin plus pemetrexed (*n* = 863). Higher response rate occurred in the cisplatin plus pemetrexed arm (28.6 %) than in cisplatin plus gemcitabine arm (22.2 %) in patients with non-squamous NSCLC. These patients had a longer OS time and trend for a longer PFS time on cisplatin plus pemetrexed (11 and 5.26 months, respectively) than on cisplatin plus gemcitabine (10.1 and 4.96 months, respectively). However, within the squamous lung cancer patients, the response rate and HR for OS as well as PFS favored cisplatin plus gemcitabine over cisplatin plus pemetrexed (Scagliotti et al. 2008, 2009, 2011).

Compared to results of Scagliotti et al., our study showed similar response rate (25.2 %) on platinum compounds and pemetrexed in non-squamous NSCLC patients. However, the duration of PFS (7 months) and OS (14 months) in our study was slightly longer than in Scagliotti et al. study. In phase III clinical trial, current and former smokers and squamous cell lung cancer patients had significantly higher risk of death compared with never-smoker and non-squamous NSCLC patients treated with cisplatin and pemetrexed. Moreover, results of phase II and III clinical trials with pemetrexed indicated factors that had statistically significant prognostic impact on survival, including gender, race, performance status and stage of disease (Scagliotti et al. 2008, 2009, 2011). In our study in Cox-adjusted analysis, we found several typical clinical factors: poor performance status, weight loss and anemia, which had affected progression and death risk. In this respect, our results were in keeping with the results of clinical trials concerning pemetrexed therapy. However, we did not observe the differences of death risk with respect to age, smoking status, gender, stage of disease and pathomorphological diagnose (adenocarcinoma vs large cell carcinoma). The impact of post study therapy on survival is difficult to evaluate because all studies permitted subsequently treatment at the discretion of the oncologists (Scagliotti et al. 2009). However, 35.7 % of our platinum plus pemetrexed-treated patients received an additional line of therapy, which resulted in prolongation of survival time to 21 months. We have to remember that these patients were in a better performance status than patients without possibility of second-line treatment.

Although the effectiveness of chemotherapy, there was a group of patients with early progression on platinum plus pemetrexed treatment (17.4 % in our study). Clinical factors are usually failed for precise prediction of treatment benefit. As for today, there are no well-defined molecular markers that are used in qualification to platinum and pemetrexed therapy in lung cancer patients. One of the best known is the relationship between the expression of ERCC1, TS, DHFR, GARFT, MTHFR as well as ERCC1 enzymes and the effectiveness of platinum and pemetrexed chemotherapy (Lee et al. 2013; Bukhari and Goudar 2013). However, the impact of the polymorphisms of genes encoding these enzymes in the efficacy of such chemotherapy is poorly described in NSCLC patients.

Li et al. (2013) had examined 45 advanced adenocarcinoma patients treated with cisplatin and pemetrexed. Median PFS was longer (6.8 vs 3.8 months) for carriers of *TS* genotypes, leading to TS expression reduction (2R/2R, 2R/3R(C) or 3RC/3R(C) in contrast to the patients with genotypes related to the high TS expression (2R/3R(G), 3R(C)/3R(G) or 3R(G)/3R(G). This *TS* gene polymorphism had predicted the response rate. However, no difference in OS was observed (10.3 vs 10.1 months, respectively). Moreover, the PFS and OS did not differ between the patients with different *MTHFR* genotypes.

In Hu et al. (2012) study, 90 advance Asian lung cancer patients received pemetrexed with platinum regimens as first-line treatment or a single-agent treatment in second line or further. The variable number of tandem repeat in 5′-UTR region of *TS* gene and deletion/insertion polymorphism in 3′-UTR region of *TS* gene was analyzed. Disease control rate, objective response rate and PFS were similar between patients harboring 2R and 3R alleles as well as alleles containing 6-bp deletion or insertion. The authors did not observe any combined effect of both *TS* polymorphisms on clinical outcome of patients treated with pemetrexed-based therapy (Hu et al. 2012).

Wang et al. (2013) revealed that Chinese patients with the deletion in both 3′-UTR regions of *TS* gene (−/− 6 bp) had significantly longer PFS and OS time than patients with insertion at least in one of 3′-UTR region (−/+ 6 bp) after short-term pemetrexed treatment (Wang et al. 2013).

In contrast to above cited studies, Arvealo et al. (Arévalo et al. 2014) had examined only 25 Caucasian patients treated with pemetrexed-based regimens. Authors showed that the presence of 3R/3R genotype significantly correlated with a superior response rate compared to presence of 2R/2R, 2R/3R and 3R/4R genotypes. Moreover, a trend toward a better PFS and significant superior OS was found among subjects showing 3R/3R genotype. Active or former smokers who were homozygous for insertion (+/+ 6 bp) in 3′-UTR *TS* region and had higher expression of *TS*, significantly more frequent responded to treatment than patients who were heterozygous +/+ 6 bp (Arévalo et al. 2014).

Polymorphisms of *TS* (both 5′-UTR VNTR and G>C polymorphism within the third VNTR) and *MTHFR* genes were tested in large study (127 Caucasian patients with complete gene polymorphisms analysis), which evaluated the effectiveness of pemetrexed plus carboplatin or pemetrexed chemotherapy. PFS was not different for patients with different *TS* genotypes. However, carriers of TT genotype in *MTHFR* gene had longer PFS time compared with patients with CC or CT genotypes of this gene (Smit et al. 2009).

Our multicenter study is one of the largest involving a homogeneous group of Caucasian, non-squamous NSCLC patients treated with front-line chemotherapy based on pemetrexed and platinum and who had simultaneous analysis of up to five different polymorphisms in *TS*, *MTHFR* and *ERCC1* genes. It should be noted that “high-expression” genotype of *TS* gene (2R/3R(G), 3R(C)/3R(G) or 3R(G)/3R(G) in opposition to “low-expression” genotype of this gene (2R/2R, 2R/3R(C) or 3RC/3R(C) had strong negative predictive value for NSCLC patients treated with platinum compounds and pemetrexed. The predictive role of *MTHFR* gene polymorphism was not clear (carriers of TT genotype of *MTHFR* gene significantly more frequently responded to chemotherapy). However, according to our results and results of Smit et al. (2009), co-occurrence of CC genotype of *MTHFR* gene and 3R allele in 5′-UTR region of *TS* gene could be assumed as negative predictive factor for pemetrexed-based chemotherapy. Whereas, *ERCC1* gene polymorphism may not predict the effectiveness of this chemotherapy. Moreover, the data concerning relation between the ERCC1 expression and/or *ERCC1* SNP and the effectiveness of chemotherapy are debatable (Friboulet et al. 2013). Therefore, the predictive value of *ERCC1* SNP for platinum-based therapy is not so obvious. It appears that the gene polymorphisms, whose expression could affect the pemetrexed’ effectiveness, are stronger predictors for this type of treatment.

Unfortunately, clinical application of polymorphism analysis in qualification to platinum and pemetrexed chemotherapy has limitation as a lack of prospective clinical trials results (e.g., JMEN). Moreover, these prospective clinical trials should contain more than one chemotherapy arms, e.g., comparison of platinum and gemcitabine chemotherapy with platinum and pemetrexed administrated according to gene signature.
